# Surface Interactions and Mechanisms Study on the Removal of Iodide from Water by Use of Natural Zeolite-Based Silver Nanocomposites

**DOI:** 10.3390/nano10061156

**Published:** 2020-06-12

**Authors:** Vassilis J. Inglezakis, Aliya Satayeva, Almira Yagofarova, Zhandos Tauanov, Kulyash Meiramkulova, Judit Farrando-Pérez, Joseph C. Bear

**Affiliations:** 1 Department of Chemical & Materials Engineering, School of Engineering and Digital Sciences, Nazarbayev University, Nur Sultan 010000, Kazakhstan; aliya.satayeva@nu.edu.kz (A.S.); almira.yagofarova@nu.edu.kz (A.Y.); 2 Environment & Resource Efficiency Cluster (EREC), Nazarbayev University, Nur Sultan 010000, Kazakhstan; 3 Faculty of Chemistry and Chemical Technology, al-Farabi Kazakh National University, Almaty 050040, Kazakhstan; zhtauanov@nu.edu.kz; 4 Department of Environmental Engineering & Management, L.N.Gumilyov Eurasian National University, Nur Sultan 010000, Kazakhstan; kuleke@gmail.com; 5 Laboratorio de Materiales Avanzados, Departamento de Química Inorgánica-Instituto Universitario de Materiales, Universidad de Alicante, 03690 Alicante, Spain; juditfarrando95@gmail.com; 6 School of Life Science, Pharmacy & Chemistry, Kingston University, Penrhyn Road, Kingston upon Thames KT1 2EE, UK; J.Bear@kingston.ac.uk

**Keywords:** natural zeolite, nanocomposites, silver nanoparticles, silver oxide, iodide removal

## Abstract

In this work a natural zeolite was modified with silver following two different methods to derive Ag_2_O and Ag^0^ nanocomposites. The materials were fully characterized and the results showed that both materials were decorated with nanoparticles of size of 5–25 nm. The natural and modified zeolites were used for the removal of iodide from aqueous solutions of initial concentration of 30–1400 ppm. Natural zeolite showed no affinity for iodide while silver forms were very efficient reaching a capacity of up to 132 mg/g. Post-adsorption characterizations showed that AgI was formed on the surface of the modified zeolites and the amount of iodide removed was higher than expected based on the silver content. A combination of experimental data and characterizations indicate that the excess iodide is most probably related to negatively charged AgI colloids and Ag-I complexes forming in the solution as well as on the surface of the modified zeolites.

## 1. Introduction 

The contamination of water resources with toxic organic and inorganic pollutants is a serious global issue. The industries that predominantly pollute the water environment include chemicals production, metal processing, textile industry and paper and pulp industry. Concerning iodide, the major source of pollution comes from nuclear power plants [1,2,3]. The produced radioactive wastes, such as isotopes of iodide (^129^I and ^131^I), are typically used for the fission of uranium [1,4,5]. Despite the robust safety level in large-scale plants there are cases in the history when processes malfuncted leading to nuclear accidents. Among them is the most recent Fukushima disaster, happened in Japan in 2011, resulted in enormous discharge of 1.5 × 10^17^ Bq radioactive isotope of iodine [4,5]. Another disaster happened in Chernobyl nuclear station in 1986 in Ukraine, resulted in the discharge of 1.76 × 10^18^ Bq of radioactive iodine [4,5]. The adverse effects caused by radioactive discharges include severe health problems, especially following inhalation and ingestion. Such poisoning can potentially damage the kidney, liver and other human organs and may progress to thyroid cancer [6]. Furthermore, they can result in long-term issues since the radioactive elements possess long half-life time and bioaccumulation [1,7,8]. Besides radioactive iodide, another source of contamination comes from the tablets used for the purification of water, which contain iodine [9,10,11]. Dissolved iodine in natural water can exist in several forms such iodide (I^−^), iodate (IO_3_^−^), hypoiodous acid (HOI), and organic iodide while in tap water and in the presence of chlorine iodide can be oxidized to iodate [12].

As pollution affects all ecosystems various remediation approaches have been employed depending on the form iodine in the environment. Among most frequently and successfully applied methods for the removal of iodide from water, are the ion exchange and membrane separation [5,13]. These methods however are relatively expensive and, in particular during ion exchange, other ions are released into the solution. An effective and relatively simple method is adsorption using various porous materials, which are typically of low cost and can be easily modified for targeted iodide removal [5,14,15,16,17]. Such low cost adsorbents are the natural zeolites, which have been studied for iodide removal from water since decades. Utilization of natural zeolites as adsorbents has attracted considerable attention due to their structural and chemical properties. These materials are cheap, non-toxic, thermally and chemically stable and thus are excellent candidates for adsorption processes. Clinoptilolite is the most known and abundant natural zeolite extensively used in water and wastewater treatment [18,19,20,21]. Clinoptilolite has been widely used in adsorption of cations from water owing to its negatively charged structure and the presence of exchangeable cations (Na^+^, K^+^, Ca^2+^ and Mg^2+^) but it has poor affinity towards anions [22]. Surfactants, organics, and noble metal doping can be used to enhance clinoptilolite affinity towards iodide [18,23,24,25,26]. Table 1 presents the studies using silver modified zeolites for the removal of iodide from water and gasses. In this case, the iodide is removed by the formation of insoluble AgI precipitate on the surface of the zeolites [3,5]. 

As shown in Table 1, there are several studies on the use of natural and synthetic zeolites for iodide removal from water, especially zeolites modified with different silver forms (Ag^0^ or Ag_2_O). All studies clearly showed the effectiveness of silver modification. Although the modification with noble metals might be considered costly, adding a small amount of silver forms into porous structures could substantially simplify the removal process by increasing the adsorption capacity, and sensitivity and reducing of resources required in comparison with other modified forms of zeolites. 

This study is a continuation of our previous research where we used fly ash-derived synthetic zeolites and cryogels doped with Ag^0^ for the removal of iodide [5,33]. Herein, natural clinoptilolite was modified with silver to produce two different nanocomposites containing metallic silver nanoparticles (Ag^0^) and silver oxide nanoparticles (Ag_2_O). Adsorption equilibrium and kinetics are presented and a detailed study of the effect of silver forms on the iodide removal along with potential mechanisms is conducted. The conclusions are supported by advanced characterization methods. To the best of our knowledge there are no comprehensive studies on different silver-modified natural zeolites for iodide removal from water and on the mechanisms involved.

## 2. Materials & Methods

### 2.1. Materials and Modification

The chemical reagents NaCl (≥99.0%), KI (≥99.0%), AgNO_3_ (>99.9%), and NaBH_4_ (99%) were purchased from Sigma-Aldrich (St. Louis, MO, USA). Ultrapure (UP) water with resistivity of 18.3 MΩ·cm was obtained by Millipore filtration (Merck, MA, US). The natural zeolite of content of 80% clinoptilolite was obtained from the company “Transcarpathian zeolitic factory” (Khust district, Ukraine) and is referred as NZU (Natural Zeolite Ukraine) in the rest of paper. Sodium chlorite (NaCl, 99.5%), sodium borohydride (NaBH_4_, 99.5%) and silver nitrate (AgNO_3_, 99.8%) used for the nanocomposites synthesis were purchased from Sigma-Aldrich (St. Louis, MO, USA). Potassium iodide (KI, 99.8%) used for the adsorption experiments was purchased from Fischer-Scientific (Hampton, NH, U).

#### Natural Zeolite Pre-Treatment and Modification

The natural zeolite sample was sieved on the vibrating sieve AS200 (Retsch, GmbH) and the particle size of 0.8–1.4 mm was used for further experiments. The zeolite was thoroughly washed with UP water and dried at 150 °C for 24 h (NZU). The pretreatment of zeolite was performed as follows: 100 g of NZU were added to 1M NaCl at 60 °C and incubated for 7 days with daily replacement of the NaCl solution. Then the material was washed with UP water for the removal of excess of NaCl and the sodium form of the zeolite (NZU-Na) was dried at 60 °C, and stored in a desiccator [34].

Two synthesis methods were followed. The synthesis of the Ag_2_O@NZU nanocomposite was performed following the method of Bo et al. [35]. First, 10 g of NZU-Na were added to 666 mL of 0.1 M AgNO_3_ solution for 48 h in dark conditions. Then the samples were washed with UP water and dried for 24 h at 60 °C. For the Ag^0^@NZU nanocomposite synthesis a method based on Lihareva et al. [34] and Tauanov et al. [36,37] was used. First, NZU-Na was converted to Ag^+^ form by ion exchange by adding 100 g of zeolite to 250 mL of 0.04 M AgNO_3_ solution in dark conditions for 24 h. Then, 13.5 g of the obtained Ag^+^ form was added in 135 mL of 50 mM AgNO_3_ solution for 48 h in dark conditions. Finally, the reduction was done by immersing the zeolite in a 0.25 M sodium borohydride solution until full coverage of the material for 1 h in cold conditions by using ice [38]. Following reduction the solution was filtered and the zeolite was dried in a bench oven at 90 °C for 8 h. Although several methods [9,39,40] of synthesizing silver nanoparticles (Ag NPs) are available, chemical reduction [39] is one of the most frequently applied methods to prepare them as colloidal dispersions in water or organic solvents. AgNO_3_ is the most commonly used precursor when preparing Ag NPs via the reduction route because of its good stability in polar solvents [9]. Sodium borohydride is one of several chemicals used as a reducing agent in the preparation of Ag NPs [11,41]. A layer of absorbed borohydride anions on the surface of the nanoparticles keeps the nanoparticles separated so to avoid agglomeration. Following drying, all zeolites were placed in closed containers and stored in a desiccator.

### 2.2. Materials Characterization

The chemical composition of the samples was studied by X–ray fluorescence (XRF) on an Axios Max (PANalytical, Malvern, UK). Measurements were done in vacuum, typically at 40–50 kV and samples were prepared as pellets with 99.5% boric acid (Sigma-Aldrich, St. Louis, MO, USA) in a ratio of 1/3. The matrix effects in the samples were adjusted by applying theoretical alpha factors and measured line overlap factors for the measured intensities. The standards that were used in the calibration procedures for analysis were the Omnian Monitor, Batch 08 from PANanalytical (Malvern, UK). X-ray photoelectron spectroscopy (XPS) spectra were recorded on a XPS K-ALPHA (Thermo Scientific Ltd., Waltham, MA, USA). All spectra were collected using an Al-K radiation (1486.6 eV), monochromatized by a twin crystal monochromator, yielding focused X-ray spot elliptical shaped with a major axis length of 400 µm at 3 mA × 12 kV. The alpha hemispherical analyser was operated at constant energy mode with survey scan pass energies of 200 eV to measure the whole energy band and 50 eV in a narrow scan to selectively measure the desired elements. Charge compensation was achieved with the system flood gun that provides low energy electrons and low energy argon ions from a single source. The CH_x_ in carbon 1s score level was used as reference binding energy (284.8 eV). The powder samples were pressed and mounted on the sample holder and placed in the vacuum chamber. Before the pattern recording, the samples were maintained in the analysis chamber until a residual pressure of ca. 5 × 10^−7^ Nm^−2^. The peaks deconvolution was performed by a quantitative analysis calculating the integral of each peak, after subtracting the S-shaped background, and by fitting the experimental curve to a combination of Lorentzian (30%) and Gaussian (70%) models. The sample mineralogy was evaluated by using X-ray diffraction (XRD) spectrometry. Patterns were recorded using a Rigaku (SmartLab^®^ X-ray, Tokyo, Japan) diffraction system with Cu Kα radiation source (λ = 1.540056 Å) at a scan rate of 0.02° θ∙s^−1^ in the 2θ range of 5–90°. The data files were obtained by X’Pert Graphics & Identify data collection software. The surface morphological characteristics of zeolites were studied by Scanning Electron Microscopy (SEM, Tokyo, Japan) using a JEOL 6380 LV scanning electron microscope, operating in LV mode, typically at 20–30 kV, equipped with a backscattered electron detector. Mapping analyses were carried out using a Si (Li) energy-dispersive x-ray spectrometer (INCA X-sight, Oxford Instruments, UK) connected to SEM. The nanoscale investigation was performed with the high-resolution JEM-2100 LaB_6_ transmission electron microscope (HRTEM, JEOL, Tokyo, Japan), operating at 200 kV. The porous structure was studied using low-temperature nitrogen adsorption measured on an Autosorb-1 (Quantachrome Instruments, Boynton Beach, FL, USA). The average pore size and total pore volume were calculated from the experimental data using the in-built software, while the pore size distribution was calculated using the BJH method.

### 2.3. Adsorption Kinetics and Equilibrium

The adsorption kinetics and equilibrium were done by bringing in contact 1 g of zeolite samples with 150 mL of iodide solutions without agitation and pH adjustment at room temperature. For kinetics experiments the concentrations of 200 ppm and 800 ppm were used and for the equilibrium experiments concentrations between 30 and 1400 ppm. Solution samples (30–100 μL) were taken after specified time and diluted up to 3 mL by using UP water before measuring the residual iodide concentration by using a UV–Vis spectrophotometer (WTW PhotoLab 6600, Xylem, NY, US) at 225–227 nm wavelength. All adsorption experiments were conducted in static (batch) conditions. Samples were taken and analyzed until equilibrium was reached, i.e., until no changes in aqueous phase concentrations were observed. The total sampling volume was kept below 5% in all experiments. The amount of iodide adsorbed was calculated as follows:

(1) $$q_{eq}=\frac{C_{o}-C_{f}}{m}\times V$$

where *q_eq_* is the iodide loading on the zeolite (mg/g), *C_o_* and *C_f_* are iodide concentrations (mg/L) in the initial and final solutions, respectively, *V* the volume of solution (L), and m is the initial weight of the zeolite (g). To avoid confusion, throughout the paper, the term “loading” and symbol *q* (mg/g) are used for the amount of species adsorbed per initial (before adsorption) weight of the solid phase and the term “content” and symbol *c_t_* (mg/g) for the amount of species adsorbed per total weight of the solid phase (initial weight plus the weight of the adsorbed species). The former is typically used for kinetics and equilibrium studies while the later for XRF, EDS, and other compositional analyses. They are related as follows:

(2) $$c_{t}=\frac{q}{1+\left(\frac{q}{1000}\right)}$$

The experiments were conducted in duplicate and the average values are presented. The average standard deviation of the solution concentration in kinetics experiments was 3.5% and in equilibrium experiments 10%. Control experiments showed that the iodide losses due to adsorption on the container walls are limited to an average of 2%.

Leaching of Ag from the zeolites was studied by analyzing the solution phase after equilibrium was reached (typically 20–30 days). Two types of measurements were performed. In the first type the zeolite was separated with a screen of 100 microns opening in order to allow formed AgI colloids and precipitates to be collected in the filtrate. Sodium triosulfate (Na_2_S_2_O_3_) was added in the filtrate to dissolve the formed AgI. In the second type, only the supernatant solutions were analysed, with and without the addition of sodium triosulfate. The Ag concentration in the solutions was analyzed by using an Atomic Absorption Spectrometer (AAnalyst 400, Perkin Elmer, MA, US). Finally, a mercury equilibrium experiment was conducted by adding 1 g of zeolite in 150 mL of mercury solution of 200 ppm concentration in static (batch) conditions at room temperature. To avoid mercury precipitation or the formation of hydrocomplexes, the initial pH was adjusted to 2 by using dilute HNO_3_. The mercury concentration was measured after 384 h by using the RA-915 M mercury analyzer (Lumex, Russia) which employs pyrolysis technique.

## 3. Results and Discussion

### 3.1. Characterization of Modified Zeolites

**Figure 1:**
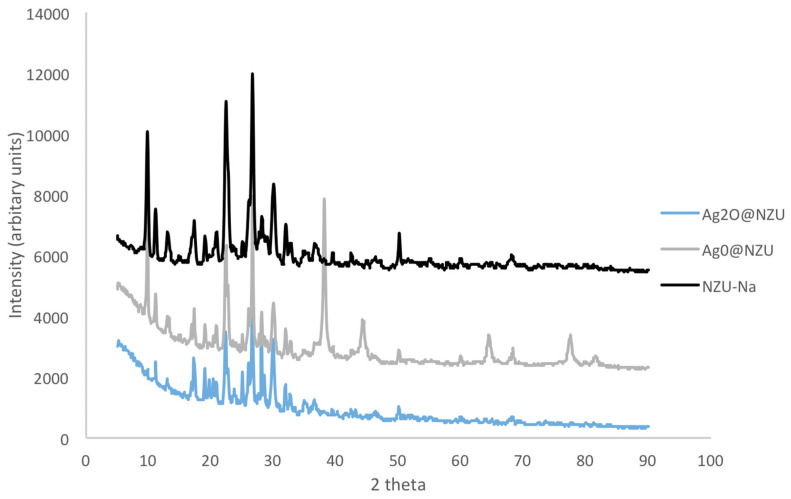
XRD patterns of Ag^0^@NZU, Ag_2_O@NZU and NZU-Na.

The mineralogical composition as determined by XRD is shown in Figure 1. NZU-Na shows the characteristic peaks of clinoptilolite at 9.8°, 22.4° and 30°. The peak at 26.6° is attributed to quartz [42]. The peaks at 38.18°, 44.3°, 64.6°, and 77.5° in the Ag^0^@NZU sample are characteristic of metallic Ag^0^. The XRD pattern of Ag_2_O@NZU sample shows no new peaks of silver oxide, probably due to masking by clinoptilolite peaks in the same positions.

**Figure 2:**
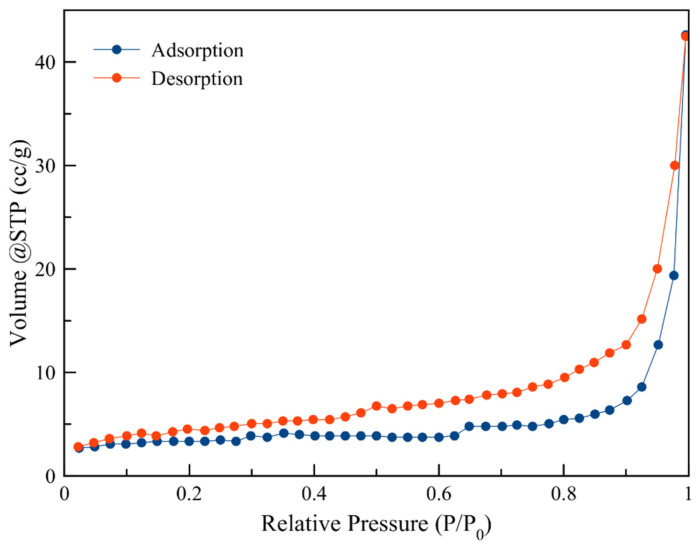
Adsorption-desorption isotherm for NZU.

The porosimetry results are presented in Table 2 and Figure 2. The hysteresis loop in the nitrogen adsorption–desorption isotherm for all samples is characteristic of mesoporous materials.

The sorption-desorption and pore size distribution indicate that both samples possess a mesoporous structure. The clinoptilolite surface area and pore characteristics are not affected considerably by the Na and Ag modification procedures. 

The chemical composition of modified zeolites clearly indicates the aluminosilicate nature of the material as shown in Table 3. The Si/Al molar ratio of the NZU sample is 5.32 as expected for clinoptilolite [42]. The NZU-Na sample is enriched in Na as expected after the modification with NaCl, and according to the results, Na^+^ it replaces Ca^2+^ from the zeolite structure. The Ag_2_O@NZU sample contains less Na, K and Ca than NZU-Na due to the exchange with Ag^+^ and the same is true for Ag^0^@NZU with the exception of Na, which is higher due to the use of sodium borohydride during reduction.

**Figure 3:**
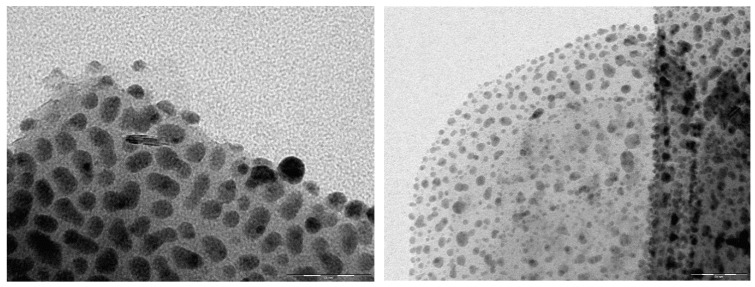

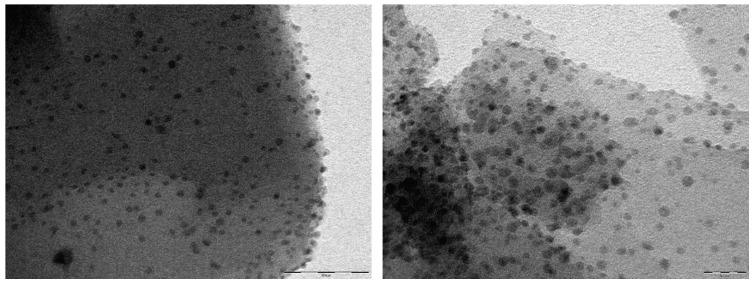
TEM images of Ag_2_O@NZU (upper left, scale 60 nm and upper right 50 nm) and Ag^0^@NZU (lower left, scale 60 nm and lower right, scale 20 nm).

The Ag^0^ nanoparticles formation, detected on XRD in all samples, was also detected by TEM imaging (Figure 3). It is obvious that in most cases the particle geometry corresponds to a well-defined spherical shape with sizes around of 5–25 nm with Ag^0^ nanoparticles generally being smaller than Ag_2_O nanoparticles. By using Scherrer equation with the three more intensive XRD peaks the estimated Ag^0^ nanoparticles size was 17.8 nm, corroborating TEM results. The existence of nanoparticles in the Ag_2_O@NZU sample shows that the ion exchanged Ag^+^ is either oxidized to Ag_2_O or it forms silver clusters. The photo-reduction of Ag^+^ species and consequent formation of Ag_m_^n+^ clusters under light exposure during Ag^+^ ion exchange has been reported for ZSM-5, NaX and NaY zeolites [43] and auto-reduction of Ag^+^ to Ag_m_^n+^ and Ag_m_^0^ has been reported for faujasite [31].

### 3.2. The Adsorption Kinetics and Equilibrium

**Figure 4:**
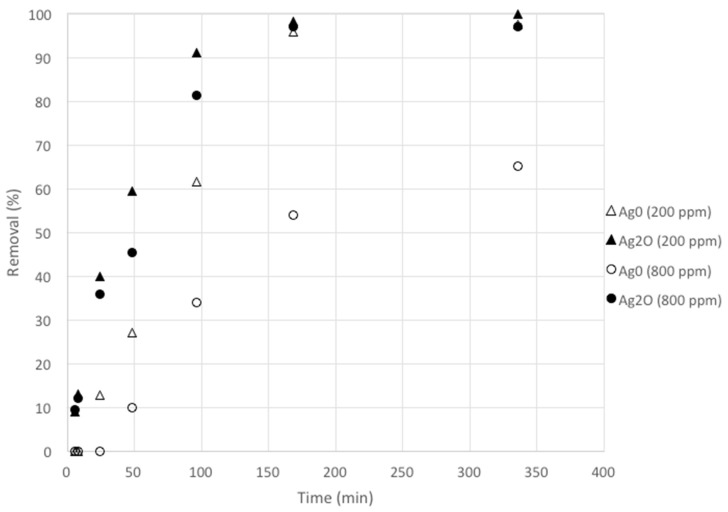
The adsorption kinetics of iodide on zeolite nanocomposites.

**Figure 5:**
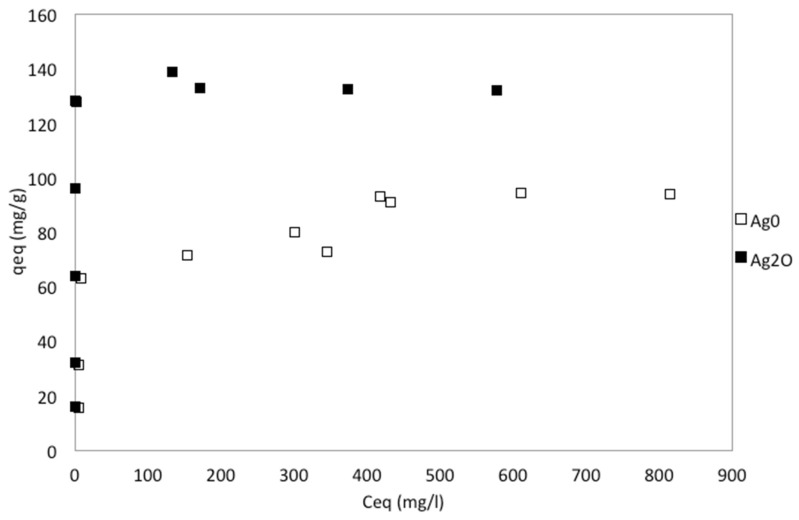
The adsorption equilibrium of iodide on zeolite nanocomposites.

The kinetic and equilibrium results are shown in Figure 4 and Figure 5. As clinoptilolite is a cation exchanger iodide is not expected to be exchanged while adsorption is difficult owing to clinoptilolite’s negative surface charge and its low to moderate surface area [42]. This is in agreement with the results as NZU showed less than 2% removal of iodide, in the range observed for the blank solutions. Faghihian et al. found that the adsorption of iodide on natural clinoptilolite is insignificant (0.25 mg/g) [28] although in a later study was found to be higher, between 4–10 mg/g [25]. Kubota et al. used a commercial zeolite which showed no affinity for iodide [44] and similar results were presented by Tauanov et al. for synthetic zeolites [5]. An exception is the work of Rehakova et al. who studied the modification of clinoptilolite of purity of 57.2% with iodide by using concentrated KI solutions in the concentration range of 0.1–1 mol/L [45,46]. The results showed that clinoptilolite can be loaded with an incredible amount of 4.21–13.45% *w*/*w* iodide. As discussed in Section 3.3, these results can be explained by the formation of crystalline I_2_ on the surface of the zeolites but given the low to moderate surface area of natural clinoptilolite the adsorption of such large amounts of I^−^ and I_2_ is questionable.

Kinetics results showed that the removal of iodide is faster when using Ag_2_O@NZU and the lower the initial iodide concentration the higher the removal rate, as expected. Kinetics and equilibrium results clearly show that the rate of adsorption and the capacity is higher for Ag_2_O@NZU. Also, equilibrium data indicate that the phenomenon is irreversible for Ag_2_O@NZU and reversible and Langmurian for Ag^0^@NZU. As formation of other iodide species can happen at low pH and oxidative conditions under certain conditions and may result in erroneous results is important to scan the solutions for identification of additional peaks. This was done during the experiments by scanning in the UV–Visible range from 190 to 400 nm and no peak other than the iodide peak was observed. IO_3_^−^ shows no peaks and the intensity is gradually decreasing from 180 nm onwards, I_2_ shows a peak at 203 nm, I^−^ has a distinctive peak at 226 nm, while I_3_^−^ shows two peaks at 288 and 352 nm [7]. It should be noted that I_3_^−^ is formed in the solution when I^−^ and I_2_ co-exist [47].

The XRF analysis of the samples before iodide adsorption (Table 3) showed that the average Ag content is about 89 mg/g for Ag_2_O@NZU and 55 mg/g for Ag^0^@NZU. Based on these values and for Ag:I molar ratio of 1 the theoretical average amount of iodide that can be removed is about 105 mg/g for Ag_2_O@NZU and 65 mg/g for Ag^0^@NZU. These values are lower than the maximum measured in equilibrium experiments, about 132 mg/g for Ag_2_O@NZU and 94 mg/g for Ag^0^@NZU. Discrepancies between expected (based on Ag content) and observed (removal experiments) on iodide solid phase loading are not uncommon and not always explained [33,48,49,50,51]. As discussed in Section 3.3, the excess iodide removed can be explained by the formation of AgI colloids and Ag-I complexes in the solution and probably on the surface of the materials.

**Figure 6:**
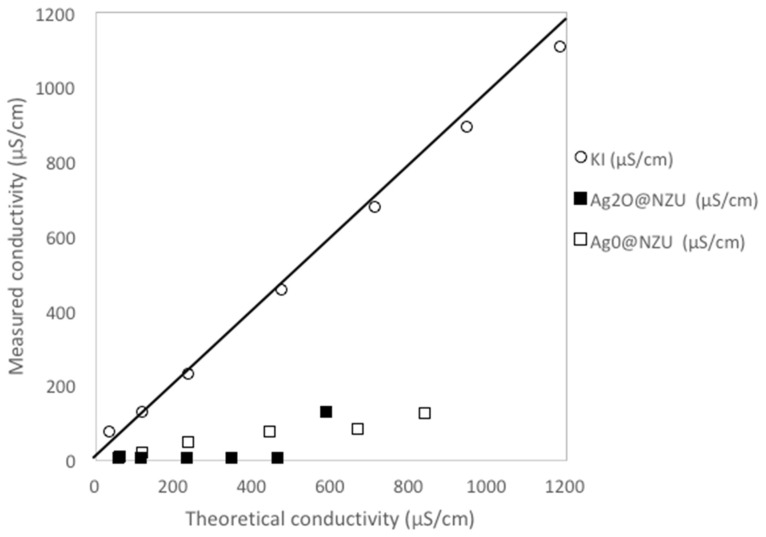
Solutions conductivity after 300 h for initial iodide concentrations of 30–1000 ppm. KI is the measured conductivity of the KI stock solutions. The limiting molar conductivities at 25 °C of I^−^ and K^+^ of 7.68 and 7.352 in mS m^2^/mol, respectively were used for the estimation of the theoretical solutions conductivity. This conductivity was calculated assuming that all K^+^ remains as free ion in the solution and no other ion besides I^−^ is removed or released in the solution.

The XRF results after the adsorption of iodide from the 800 ppm solution are shown in Table 4 and they confirm the presence of I^−^ on the surface of the zeolites and that loading is higher in the Ag_2_O@NZU sample but the iodide amount is considerably lower than this measured in the equilibrium experiments. Several XRF iodide measurements were done in the framework of the present study and the iodide content was always underestimated. Taking into account the large number and the consistency of equilibrium experimental data, it is the authors’ opinion that XRF results for iodide can only be used as rough approximations and, as discussed below, iodide losses during characterizations cannot be excluded. Also, there is a significant increase of the K content of the zeolites. This can be related to the considerable decrease of conductivity in all solutions beyond levels that can be explained by the removal of iodide alone (Figure 6). The measurements showed that the solutions conductivity after iodide removal is only 1–22% of the theoretical conductivity. The removal of K^+^ from the solution can be attributed to occlusion into the AgI precipitate or, as discussed in Section 3.3, to the adsorption on negatively charged AgI colloids or Ag-I complexes.

The porosimetry results do not show any significant changes besides the decrease of the total pore volume of the Ag_2_O@NZU and the increase of the average pore size of Ag^0^@NZU (Table 5). These changes can be attributed to the blockage of the zeolite pores due to the formation of AgI precipitate, but the data are not clear enough to draw safe conclusions.

**Figure 7:**
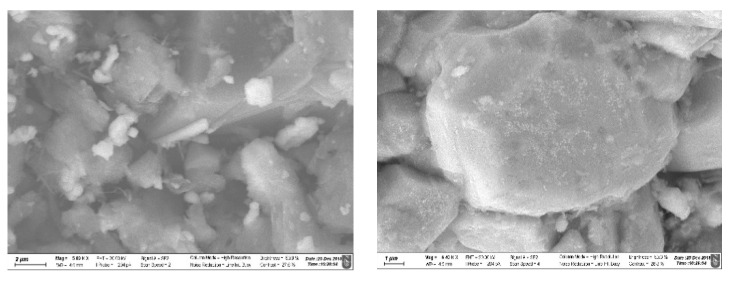
SEM images of Ag_2_O@NZU (**left**) and Ag^0^@NZU (**right**) samples (scale 1-2 μm).

**Figure 8:**
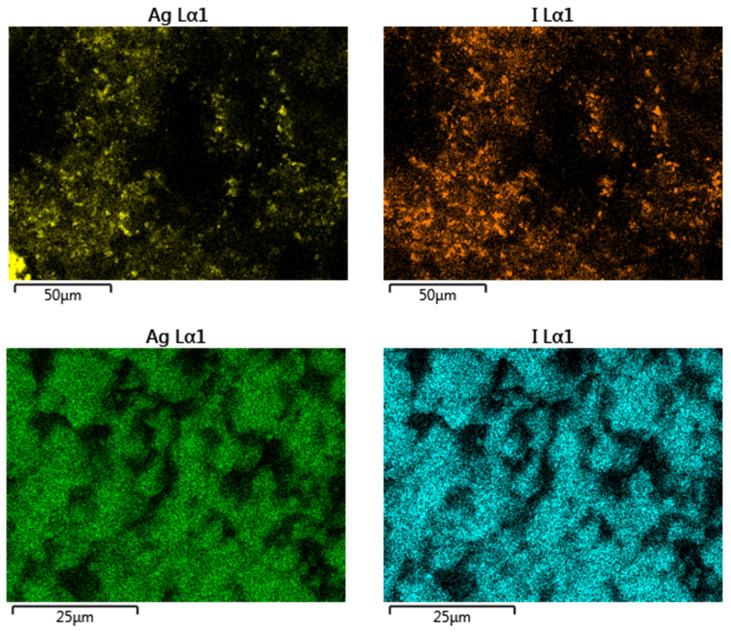
SEM/EDS mapping images of Ag_2_O@NZU (**upper**) and Ag^0^@NZU (**lower**) samples.

The surface morphology of modified zeolites after adsorption of iodide from the 1000 ppm solution was studied using SEM analysis (Figure 7). SEM images clearly show that Ag and I are found on the same spots and at high magnification crystals of a precipitate are covering the surface of the materials, presumably AgI. In total four SEM/EDS elemental mapping measurements over areas from 1.5 × 1.5 μm to 150 × 150 μm for each sample were performed and the results showed that the Ag:I molar ratio is 1.22 ± 0.03 for the Ag_2_O@NZU sample and 1.32 ± 0.20 for the Ag^0^@NZU sample (Figure 8). Based on the equilibrium results for this concentration the samples have reached saturation (Figure 5). This means that some Ag is not reacting, corroborating the XRD results, which show that some amount of Ag^0^ remained on the surface of the Ag^0^@NZU sample (Figure 10). However, the major reason of lower than expected iodide content detected by SEM/EDS may be due to losses during characterization. For instance, AgI losses during XRD and SEM/EDS characterizations were observed in a study of methyl iodide adsorption on a silver ion-exchanged ZSM-5 and they were attributed to detachment of the precipitate particles from the surface of the zeolite [30]. Also, AgI decomposes under light, high energy electron beams, as in TEM analysis and in any other environment where temperature is above 150 °C [23,52,53,54]. If this happens, I_2_ is formed and evaporates according to the following reaction:

AgI → Ag^0^ + I_2_ (R1)

**Figure 9:**
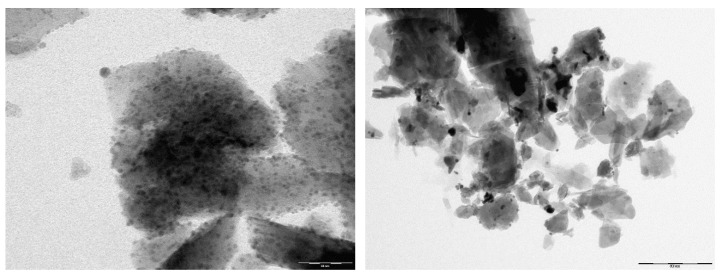
TEM images of Ag_2_O@NZU ((**left**), scale 50 nm) and Ag^0^@NZU ((**right**), scale 500 nm) samples.

TEM analysis performed on the samples after iodide adsorption from the 200 ppm solution showed less nanoparticles and dark areas as a result of the surface coverage with the precipitate (Figure 9). TEM/EDS analysis of individual Ag nanoparticles after the iodide adsorption showed very low iodide content, confirming the AgI decomposition under high-energy electron beams.

**Figure 10:**
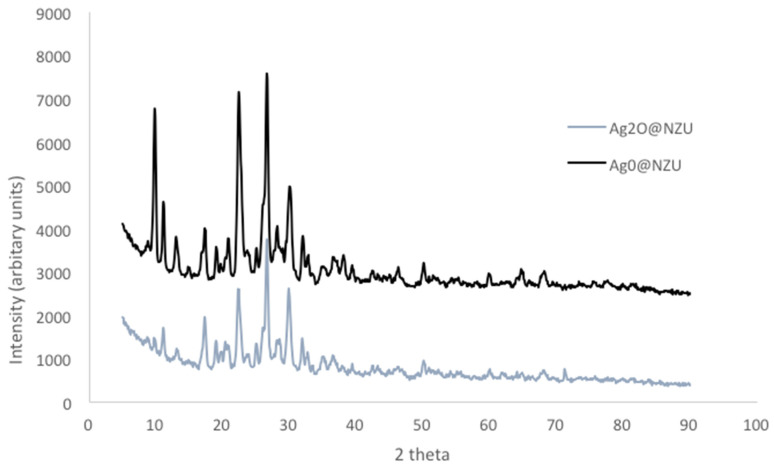
XRD pattern of modified zeolites after iodide adsorption.

The formation of AgI precipitate on the surface of the material was not confirmed by XRD analysis (Figure 10). This can be explained by the masking of AgI peaks by zeolite peaks at 22.39°, 23.68°, 39.40°, 42.68°, and 46.4°. The only difference between the Ag_2_O@NZU and Ag^0^@NZU samples is the Ag^0^ peak at 38.1°. Also, the other Ag^0^ peaks disappeared, a result of the oxidation by dissolved oxygen and the interaction with iodide.

**Figure 11:**
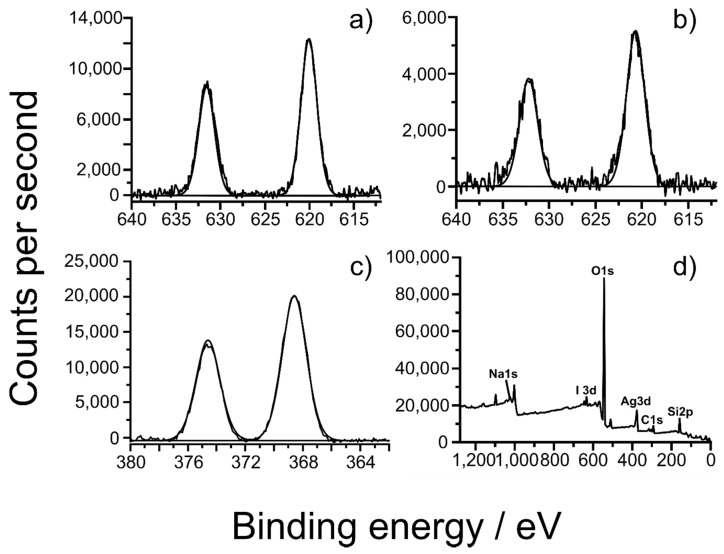
XPS results after interaction with iodide. Figures show: high resolution scans of (**a**) I3d of Ag^0^@NZU at 200 ppm I^−^, (**b**) I3d of Ag_2_O@NZU at 800 ppm I^−^, (**c**) Ag3d of Ag^0^@NZU at 800 ppm I^−^ and (**d**) Ag^0^@NZU at 200 ppm I^−^ survey.

A detailed discussion is dedicated to XPS analysis as is important for deriving the oxidation state of the silver and iodide on the zeolite surface. The results are presented in Figure 11 and Table 6. The expected the 1s peak of Na_2_O is at 1072.5 eV, close to this of the molecular sieves A, X, and Y which is in the range of 1071.5–1072.6 eV [55]. Two oxidation states were observed for silver before iodide adsorption and in combination to XRD and TEM results they most probably belong to Ag_2_O and Ag^0^. After the iodide adsorption one silver oxidation state is detected, presumably Ag^+^ resulting from the formation of AgI. According to the NIST database, the 3d_5/2_ peak of Ag^0^ is in the range of 367.9–368.3 eV and of Ag_2_O in the range of 367.7–368.4 eV [55]. After the adsorption of iodide one oxidation state of iodide is detected at 619.9-620.6 eV, which is either I^−^ or I_2_. The expected 3d_5/2_ peak of I^−^ in AgI is in the range of 619–619.5 eV and of I_2_ in the range of 619.9–620.8 eV [55,56]. However, 3d_5/2_ peaks of iodide at 619.3–619.9 and even as high as 623.5 eV have also been reported [56,57,58]. The 3d_5/2_ peaks of Ag^0^ in the Ag^0^@NZU are only slightly shifted to higher value and are close to the reported values. The rest of Ag peaks and the 1s peak of Na_2_O are shifted to higher values, especially those of the zeolites before iodide adsorption. Taking these shifts into account, the 3d_5/2_ peaks in the range of 619.9–620.6 can be attributed to I^−^. In general, the higher oxidation states of iodine the higher the binding energy [58]. Thus, if iodide becomes more electropositive is possible to show a shift to higher binding energy. These shifts to higher binding energy can be explained by the effect of the zeolite matrix as discussed below.

As is well-known, besides chemical shifts, XPS peak positions are affected by the local electrostatic environment induced by the surface of the substrate and/or the surrounding polar groups [59,60]. Large differences in electronegativity between the elements of a solid are sometimes responsible for shifts in the binding energy and a very electronegative element shifts the binding energy of neighboring atoms to higher values [61]. These shifts are superimposed on the commonly observed chemical shifts and can easily be in the range of 1 eV [62,63]. It is noteworthy that zeolitic materials containing metal dopants are much studied using XPS in the literature, but consensus on how metal dopants/additives behave is elusive. XPS analysis of zeolites is particularly challenging due their poor electrical conductivity and surface inhomogeneity. As a result, the range of chemical shifts for most elements is small compared to the uncertainties in the determination of the values [64] and thus literature data can only be considered as trend indicators. For example, Gunter et al. studied chabazite and found pronounced shifts for the Fe 2p_3/2_ peak at 714.6 eV compared to 709.9–711.6 eV for Fe_2_O_3_ and the Cu 2p_3/2_ peak at 936.7 eV compared to 932.7–934.6 eV for CuO [55,65]. Ruiz-Serrano et al. studied clinoptilolite and the Ca 2p_3/2_ was shifted to 348.3 eV compared to 346.1–347.3 eV for CaO and the Fe 2p_3/2_ peak was shifted to 713.3 eV [55,66]. The authors concluded that the shift in the energy binding of elements in solids such as zeolites can be attributed to the chemical environment of the matrix and are correlated with the Si/Al ratio. Chmielewska et al. studied nano-FeO(OH) modified clinoptilolite and found a shift of Fe 2p_3/2_ at 713 eV [55,67]. This shift was attributed to the effect of Al atoms of the zeolite structure. Rehakova et al. studied iodide forms of clinoptilolite and identified 3d5/2 peaks at 619.3 eV and 621.1 eV attributed to I- and I2, respectively [45]. Panayotova et al. studied a silver modified clinoptilolite and the 3d5/2 Ag0 peak at 369.3 eV [68]. The authors state that this shift to higher binding energy could be due to the small size of silver nanoparticles. Borko et al. studied clinoptilolite loaded with Pd and found that the presence of PdO2 is indicated by the Pd 3d5/2 peak at 339 eV binding energy compared to 337.9 eV reported for PdO2 [55,69]. Zhai et al. found that the binding energy of the guest in the zeolite host-AgI composite materials, such as silver and iodine elements, shifted to higher energies [56]. For instance, the binding energy of I3d_5/2_ level moved from 619.5 eV for bulk silver iodide to 619.9 eV for the NaZSM-5–AgI composite. An exception to the studies above is the shift to lower binding energy reported by We et al. [70] who studied a clinoptilolite/mordenite sample and after modification with Na^+^ a new peak for Na1s at a binding energy of 1060.1 eV was identified.

Therefore, in our study, the Ag3d_5/2_ peak shifts to higher binding energy, is probably attributed to sample charging and the Si of the zeolite matrix. This is supported by a recent paper highlighting the inaccuracy of XPS calibration in charging samples, particularly those which are not electrically conductive such as zeolites [71]. The iodide high resolution scans are more eloquent that these of ssilver, indicating the strong possibility of the presence of I^−^ with single environment and binding energies for the I3d_5/2_ in the 619.9–620.6 eV range, attributable to I^−^.

### 3.3. Removal Mechanism and Surface Interactions

The discrepancy between the expected (based on the Ag content) and measured (equilibrium results) I^−^ content and the XPS I3d_5/2_ peak location could be explained by the formation and adsorption of I_2_ on the surface of zeolites. Mao et al. observed the same discrepancies on I^−^ content when working with Ag@CuO_2_ nanoparticles [48]. They found that besides the formation of AgI the oxidation of I^−^ to I_2_ takes place in the presence of O_2_ in the solution followed by adsorption of I_2_ on the surface of the nanoparticles. XPS analysis showed that the I3d_5/2_ peak can be divided into four different peaks at 618.4, 618.9, 619.4 and 619.7 eV, attributed to the peaks of NaI, CuI, AgI, and I_2_, the later having significantly smaller area indicating a small amount of I_2_. Li et al. also identified a small fraction of I_2_ species residing at 620.3 eV oxidized by dissolved O_2_ [72]. These studies are not clear on the possibility of I_2_ formation in the solution but given the low I^−^ concentration used, the slightly acidic pH and in the absence of strong oxidant the formation of significant amounts of I_2_ in the solution is unlikely. Also, it is not clear why the oxidation of I_2_ by O_2_ happens only on the surface of the particles. Similar results were presented by Liu et al., however they used Ag_2_O-Ag/TiO_2_ materials under visible light irradiation [73]. They found that I^−^ is photocatalytically oxidized to I_2_ and then it is adsorbed on AgI to form AgI_3_ and AgI_2n+1_ complexes and, as a result, only I^−^ attributed to AgI was detected by XPS. The formation of AgI_3_ is discussed by Chen et al. who used Ag_2_O-Ag_2_O_3_ ZIF-8 composite but the oxidation of I^−^ is caused by the reduction of Ag^3+^ to Ag^+^ and the formation of AgI [16]. Rehakova et al. studied the modification of clinoptilolite with iodide by using concentrated KI solutions and XPS showed two peaks at I3d_5/2_ 619.3 eV and 621.1 eV attributed to I^−^ and I_2_, with I^−^/I_2_ ratio of 3.9 [45,46]. According to the authors, the oxidation is happening on the surface due to a redox reaction with the Fe^3+^ of the zeolite and the produced I_2_ is adsorbed. A study conducted in the framework of the present study by using Medusa software showed that the aqueous phase speciation of iodide solutions in a mild oxidative environment of 0.6 V and in acidic to neutral solutions at 0.001 mol/L only I^−^ exists, at 0.01 mol/L a small amount of about 5% I_3_^−^ is formed, at 0.1 mol/L the solution contains almost 50% I_3_^−^ and at 1 mol/L almost 24% I_3_^−^ and 68% of crystalline I_2_ coexist. Thus, taking into account that, in the presence of iodide and triiodide, iodine is formed [74] in concentrated iodide solutions, the formation of I_2_ is plausible even without the redox reaction with Fe^3+^.

The results of the present study show that iodide is not removed by NZU and UV-Vis scans of the blank and other solutions showed no other species than I^−^. Thus, the dissolved O_2_ and Fe^3+^ induced oxidation of I^−^ on the surface of the zeolite can be excluded. Another possibility is the redox reaction between I^−^ and Ag_2_O leading to I_2_ and Ag^0^, however such a reaction has not been observed in other studies and there is no strong evidence of this reduction in XRD results (Figure 11). Also, the reduction of Ag_2_O to Ag^0^ should be hindered by the presence of I^−^ and the formation of AgI. Indeed, regardless the formation of I_2_ or other products, the formation of AgI in aqueous solutions when iodide interacts with silver has been confirmed in several studies and different materials [4,5,14,15,33,35,75,76,77,78] including clinoptilolite [26,28,29]. Also, the interaction of Ag and I_2_ in aqueous solutions results in the formation of AgI as well [79]. The interaction of iodine with silver has been extensively studied in vapor-solid adsorption and can be a complex phenomenon leading to I_2_ adsorption and several charged AgI forms [80,81]. Moreover, in the occasion of significant amounts of I_2_, XPS should have detected two peaks at I3d_5/2_ indicating the coexistence of both oxidation states of iodide. Although unlikely, in the occasion of I^−^ reduction to I_2_ on the surface of the zeolite the following reaction can take place [16]:

AgI + I_2_ → AgI_3_ (R2)

**Figure 12:**
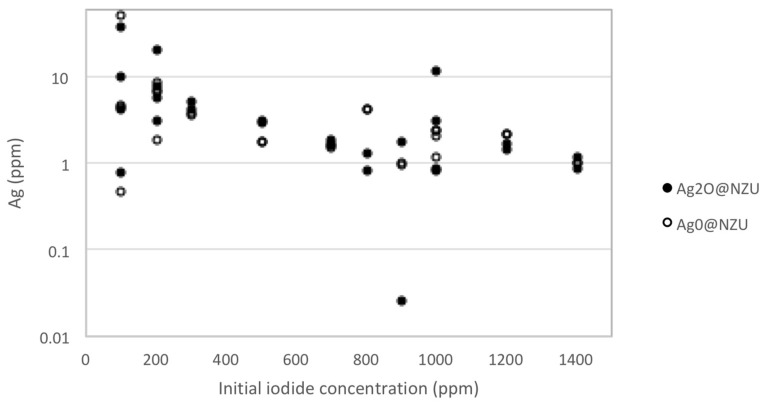
Ag leaching results (52 experiments).

The AgI_3_ gives a single environment (presumably I^−^) peak on XPS [16]. Without excluding a contribution from the adsorbed I_2_ or the formation AgI_3_, the hypothesis in the present work is that the discrepancy between the expected (XRF) and measured (equilibrium) I^−^ content can be explained by the formation of AgI colloids in the solution and on the surface of the zeolites. The Ag leaching experiments showed that the addition of oxidant results in an increase of silver concentration in the solutions, indicating the existence of some external precipitation of AgI. Nevertheless, of the 52 experiments, only four showed silver concentration higher than 10 ppm and most of measurements were around the average of 5 ppm (Figure 12). The average concentration of Ag the solutions corresponds to about 0.75 mg of leached Ag per gram of zeolite or less than 0.8% and 1.5% of the Ag content of the Ag_2_O@NZU and Ag^0^@NZU, respectively.

**Figure 13:**
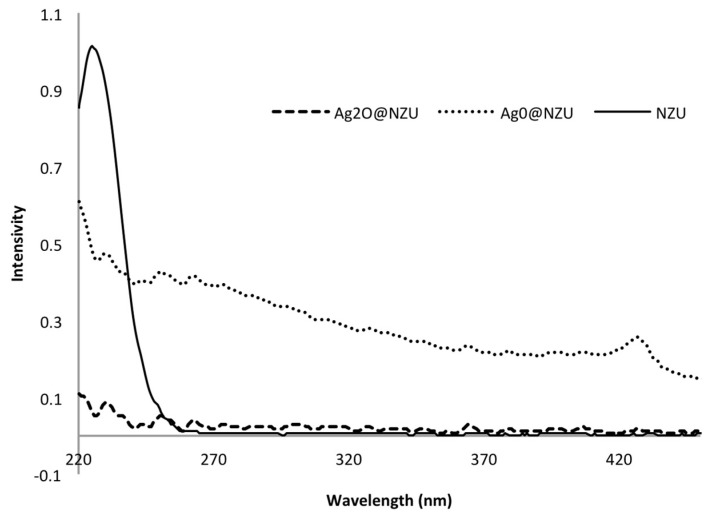
UV-Vis scan after 72 h of adsorption at 200 ppm iodide.

As Baimenov et al. discussed, when silver ions are released in a solution of excess of iodine ions negatively charged AgI colloids are formed which exhibit Ag:I molar ratios lower than 1 [33]. Indeed, after the iodide adsorption the color of some solutions turned light yellow and UV-Vis scans showed a peak at around 424–427 nm, which is attributed to AgI colloids (Figure 13). The formation of AgI colloids and appearance of the UV-Vis peak at 424–427 nm was not systematic, i.e., depends on time and concentration, and is more likely in higher iodide concentrations. Also, as is clear from the NZU UV-Vis scan, no other peaks than iodide were observed (Figure 13). However, the leached amount of silver does not seem enough to account for the excess of iodide removed from the solution thus we postulate the formation of negatively charged AgI colloids or Ag-I complexes on the surface of the zeolites. Also, the formation of colloids and/or complexes can explain the removal of K^+^ from the solution it can be adsorbed on the negatively charged Ag-I complexes or AgI colloids to form the Stern layer. Notably, other studies have shown that the otherwise insoluble AgI can be partly emulsified into small clusters if sufficient excess iodide ions are present [82,83]. These clusters contain AgI pairs and excess of iodide ions and are surrounded by an electrolyte solution rather than being part of a lattice [83]. The deposition of colloids from the water on solids surfaces is a well-known phenomenon [84], but further analysis on the deposition or formation of colloids on the surface of solids is beyond the scope of the present study.

The differences between the Ag_2_O@NZU and Ag^0^@NZU samples in terms of removal rate and maximum capacity can be explained by the difference in Ag content. However, another decisive factor is the reaction mechanism, which favors the Ag_2_O@NZU sample. The reactions are as follows [33]:

4Ag^0^ + O_2_ → 2Ag_2_O (R3)

Ag_2_O + 2H^+^ → 2Ag^+^ + H_2_O (R4)

Ag^+^ + I^−^ → AgI (R5)

Ag^+^ + nI^−^ + mK^+^→ [(AgI_n_)^1−*n*^K*^m^*]^(*m*+1)−*n*^ (R6)

The precipitation reaction R5 requires Ag^+^ and thus Ag^0^ has to be oxidized first following reactions R3 and R4. This will impede the removal of iodide by Ag^0^@NZU resulting in slower kinetics and, if oxidation is incomplete, to lower capacity, explaining the kinetics (Figure 4) and equilibrium results (Figure 5). As the reaction proceeds, an AgI layer forms below the Ag_2_O layer through which oxygen and iodide involved in the reaction diffuse. The mechanism was described by Krausmann and Drossinos, who used bulk silver (2 μm) to react with iodide and iodine in aqueous solutions [79]. In addition, they argued that the AgI layer is non-porous, since the molar volume of the iodide is greater than metallic silver by a factor of 2.5. This means that the size of Ag nanoparticles affects the overall removal rate. The proposed mechanism can possibly explain the Langmuir isotherm for Ag^0^@NZU as the overall removal depends on three rather one reaction. Also, the pH of the solutions was increased from about 6 for KI to about 7.6 for Ag_2_O@NZU and almost 10.4 for Ag^0^@NZU. Reaction R4 can explain the increase of pH but in the case of Ag^0^@NZU there is no obvious reason for such a considerable pH increase and more experiments are needed to study this effect. Finally, reaction R6 leads to the formation of colloids in the solution and on the surface of the zeolites providing a reasonable explanation of the excess iodide found in equilibrium experiments and the K^+^ removal from the solution. Also, as the I^−^ is loosely bounded on these complexes and colloids they can be easier lost under high-energy radiation, especially when characterizations are done in vacuum as is the case of XRF, SEM/EDS and TEM/EDS.

To further illustrate the effect of silver form on the removal mechanism, mercury removal experiments were conducted and the results showed a reverse in the achieved capacities in comparison to iodide removal. In particular, the maximum loading for Ag^0^@NZU was about 29 mg/g almost double than this of Ag_2_O@NZU, which was about 14 mg/g. Reactions R3 and R4 take place along with the following [85]:

Ag^0^ + Hg^2+^ → Ag^+^ + Hg^+^ (R7)

Ag^+^ + Cl^−^ → AgCl (R8)

2Hg^+^ + 2Cl^−^ → Hg_2_Cl_2_ (R9)

Reaction R7 requires the presence of Ag^0^ and this is the reason of the higher mercury removal by Ag^0^@NZU. Also, Ag_2_O@NZU sample removes some mercury owing to some Ag^0^ in its structure as XPS analysis showed (Table 6 and Figure 11). Thus, it is the silver content combined to the reaction mechanism that can favor one silver form over another. Nevertheless, more experiments are needed in order to better understand the interplay between aqueous and solid phase chemistry.

## 4. Conclusions

A natural zeolite with 80% clinoptilolite content was modified with silver following two different methods. XRD, XRF, XPS, and TEM characterizations showed that the derived zeolites are decorated with Ag_2_O and Ag^0^ nanoparticles of size of 5–25 nm and the silver content is between 55 and 89 mg/g. The materials were used for the removal of iodide from aqueous solutions in a wide range of initial concentrations, i.e., from 30 to 1400 ppm. While the natural zeolite shows no affinity for iodide, silver forms can remove up to 132 mg/g iodide. The study of the removal mechanism is challenging and the kinetics and equilibrium results along with XRD, XRF, SEM/EDS, XPS, and TEM/EDS characterizations were used to interpret the phenomena that take place on the surface of the zeolites. The experimental data showed that AgI precipitate, AgI colloids, and Ag-I complexes are formed in the solutions and possibly on the surface of the materials. Based on these observations, a set of probable reactions is provided.

## Figures and Tables

**Table 1:** Natural and synthetic silver modified zeolites applied for removal of iodide/CH_3_I from water and gasses.

Major Phase ^1^	Silver form	I^−^/I_2_, (ppm)	Initial pH	m/V (g/L)	Adsorption Capacity (mg/g)	Ref.
**Water phase (I^−^)**						
Analcime/ Sodalite (S)	Ag^0^	450	2.5	5	20.44	[5]
Ag-Z (S)	Ag^+^	10	6.2	5	1.9	[27]
Clinoptilolite/ natrolite	Ag^+^	1270–12,700	3–10	5	146/52	[28]
Clinoptilolite	Ag^+^	381–762	6–7	-	89	[26]
Clinoptilolite	Ag^+^	10^−5^ Ci/l ^2^	-	-	178	[29]
**Gas phase (I_2_)**						
ZSM 5 (S) ^3^	Ag^+^	2000	-	-	0.05	[30]
Faujasite ^3^	Ag^0^	1333	-	-	223	[31]
Modernite	Ag^+^/Ag^0^	-	-	-	-	[32]
Faujasite ^3^ Modenite ^3^ Ferrierite ^3^ ZSM 5 (S) ^3^						
BETA zeolite (S) ^3^	Ag^+^	1333	-	-	46–267	[23]

^1^ S: synthetic. ^2^ Radioactive ^131^I was used and the concentration is expressed as activity. ^3^ Methyliodide was used in gas phase.

**Table 2:** BET/BJH analysis of zeolite samples.

Sample	Surface Area (BET, m^2^/g)	Total Pore Volume (BJH, cm^3^/g)	Average Pore Size (BJH, nm)
NZU	12.6	0.027	5.82
NZU-Na	12.5	0.032	2.98
Ag_2_O@NZU	8.70	0.061	5.42
Ag^0^@NZU	14.0	0.034	2.97

**Table 3:** XRF analysis results of zeolite samples (% *w*/*w*).

	NZU	NZU-Na	Ag_2_O@NZU	Ag^0^@NZU
Na_2_O	1.13 ± 0.01	1.94	1.08 ± 1.32	2.42 ± 1.34
MgO	0.33 ± 0.06	0.27	0.41 ± 0.18	0.41 ± 0.15
Al_2_O_3_	12.72 ± 0.33	12.70	11.67 ± 0.37	12.06 ± 0.38
SiO_2_	76.55 ± 1.10	78.52	70.97 ± 1.74	72.84 ± 2.08
K_2_O	3.22 ± 0.33	3.16	2.20 ± 0.18	2.45 ± 0.26
CaO	2.20 ± 0.29	0.82	0.44 ± 0.06	0.52 ± 0.03
Fe_2_O_3_	1.60 ± 0.05	1.49	1.40 ± 0.26	1.41 ± 0.20
Ag_2_O	-	-	9.58 ± 1.40	5.92 ± 3.55

**Table 4:** XRF analysis results of zeolite samples after iodide adsorption (major elements only, % *w*/*w*).

	Ag_2_O@NZU	Ag^0^@NZU
Na_2_O	0.4	1.37
MgO	0.69	0.63
Al_2_O_3_	11.60	12.00
SiO_2_	65.60	68.50
K_2_O	6.94	7.18
CaO	0.35	0.41
Fe_2_O_3_	1.29	1.21
Ag_2_O	8.73	5.78
I	3.97	2.27

**Table 5:** BET analysis of zeolite samples after iodide adsorption.

Sample	Surface Area (BET, m^2^/g)	Total Pore Volume (BJH, cm^3^/g)	Average Pore Size (BJH, nm)
Ag_2_O@NZU	11.9	0.035	3.73
Ag^0^@NZU	10.5	0.028	9.02

**Table 6:** XPS binding energy results (eV).

	Ag_2_O	Ag^0^	Ag_2_O (200 ppm I^−^)	Ag^0^ (200 ppm I^−^)	Ag_2_O (800 ppm I^−^)	Ag^0^ (800 ppm I^−^)
**C1s Scan A**	284.6	284.6	284.6	284.6	284.6	284.6
**Ag3d_5/2_ Env. A**	369.2	368.5	370.4	368.6	369.1	368.5
**Ag3d_5/2_ Env. B**	370.4	369.7	-	-	-	-
**I3d_5/2_ Scan A**	-	-	620.6	620.0	620.6	619.9
**Na1s Scan A**	1073.2	1073.1	1072.7	1072.9	1072.7	1072.8
